# Self-care nursing assessment: cross-cultural adaptation and validation of the Spanish version of the Self-care of chronic illness inventory

**DOI:** 10.1186/s12912-023-01605-1

**Published:** 2023-11-22

**Authors:** Noelia Durán-Gómez, Casimiro Fermín López-Jurado, Miguel Ángel Martín-Parrilla, Jesús Montanero-Fernández, Demetrio Pérez-Civantos, Macarena C. Cáceres

**Affiliations:** 1 Departamento de Enfermería, Universidad de Extremadura, Facultad de Medicina y Ciencias de La Salud, 06006 Badajoz, Spain; 2 Departamento de Enfermería, Universidad de Extremadura, Centro Universitario de Plasencia, 10600 Plasencia, Spain; 3 Departamento de Matemáticas, Universidad de Extremadura, Facultad de Medicina y Ciencias de La Salud, 06006 Badajoz, Spain; 4 Facultad de Medicina y Ciencias de La Salud, Universidad de Extremadura Hospital Universitario de Badajoz, 06006 Badajoz, Spain

**Keywords:** Nursing, Self-care, Chronic illness, Cultural assessment, Psychometrics, Validity

## Abstract

**Background:**

Self‐care is the primary means of caring for a chronic condition. Therefore, it is necessary to assess it by using a good validity and reliability instrument. The Self-Care of Chronic Illness Inventory (SC-CII) is a generic instrument developed to measure self-care processes behaviors using three separate scales in patients with chronic illness. The original cross-cultural assessment concluded the need for future studies sampling patients from different sites to increase the generalizability of the psychometric evaluation results. It was unclear whether this tool had sound psychometrics properties in the context of Spanish culture. The purpose of this study was to cross-culturally adapt the SC-CII, test its psychometric properties and validate its use among Spanish people with chronic diseases.

**Methods:**

A cross-cultural translation of the SC-CII was performed from English to Spanish. The psychometric evaluation was conducted in a sample of 350 patients with chronic conditions through a multicenter cross-sectional study based on the STROBE guideline. Data were collected from face-to-face interviews during 2022. Internal validity was assessed with Confirmatory Factor Analysis, internal consistency reliability with Cronbach alpha for unidimensional scales and McDonald's Omega reliability coefficient for multidimensional scales.

**Results:**

Most (63.4%) participants were older adults aged 65 years or older with a mean age of 65.45 ± 14.97. The average number of chronic conditions reported was 2.81%; the most common conditions were hypertension (52.3%), musculoskeletal disorders (46.3%) and diabetes (38.9%). Patients reported adequate self-care behaviors in all three scales of the SC-CII. The Self-Care Maintenance and Management scales were multidimensional, and the Self-Care Monitoring scale was unidimensional. In Confirmatory Factor Analysis, the Self-Care Maintenance and Monitoring scales had satisfactory fit indices. The Self-care Management scale had acceptable fit indices. The Omega reliability coefficient for multidimensional scales was 0.75 (Self-Care Maintenance) and 0.72 (Self-Care Management). The Cronbach alpha coefficient of the Self-Care Monitoring scale was 0.85. Item-total correlations were all significant except one. Test–retest reliability showed an intraclass correlation coefficient of 0.92.

**Conclusions:**

The SC-CII has appropriate psychometrics characteristics and is a culturally suitable and reliable instrument for assessing to the self-care behaviors of patients with chronic disease in Spain. The scale provides a simple and rapid solution to assess the self-care process.

## Background

Nowadays, the leading cause of death in the world is non-communicable diseases (NCDs) or chronic diseases, responsible for 74% of deaths worldwide. This group includes cardiovascular diseases, cancer, diabetes and chronic respiratory diseases, among others [1].

In the Progress Monitor 2022 report [1], the World Health Organization considers that NCDs involve key risk behaviors that are central to the origin of certain diseases, such as obesity, high blood pressure or hypercholesterolemia, which are based on modifiable risk factors. These factors, which include sedentary lifestyles, high alcohol and tobacco consumption, unhealthy diet and their associated diseases, are currently a major global public health challenge, especially in low- and middle-income countries, as they are responsible for about three-quarters of NCD deaths.

Chronic diseases share two fundamental characteristics. On the one hand, they represent the main threat to health in all healthcare settings and, on the other, they are a factor generating high healthcare costs. Within this framework, all health systems are reformulating their interventions to focus on the prevention and control of NCDs. In this context, the importance of self-care is increasingly recognized [2]. To counteract the burden of chronic illnesses, healthcare systems and providers must promote patient self-care.

Different research has proposed that mastery of self-care is the key element in the management of chronic diseases [3]. Without it, it is impossible to manage them. Self-care is the primary means of caring for chronic diseases because it provides the necessary behaviours that lead the patient to maintain stability and control their symptoms [2]. In chronic illness, higher levels of self-care have been associated with better health outcomes [4, 5] including decreased hospitalization, costs, and mortality [6, 7].

**Fig. 1:**
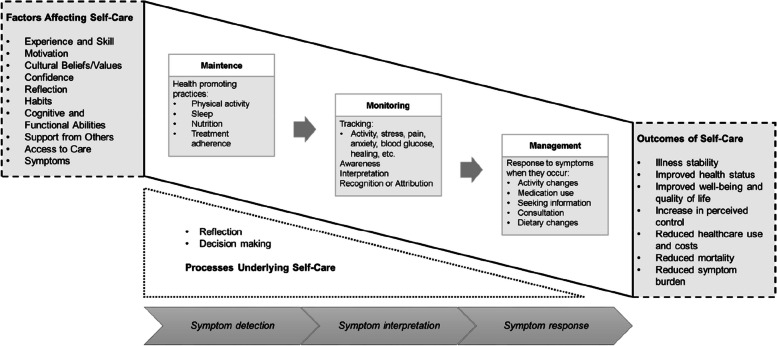
Conceptual framework of the Mid-Range Theory of Chronic Disease Self-Care integrating symptoms with self-care[9].

In 2018, Riegel et al. [2] developed a 20-item self-report instrument based on the Mid-Range Theory of Chronic Illness Self-Care [8], which provided the framework for the original instrument. This theory defined self-care as a complex and dynamic process performed throughout life of maintaining health through health-promoting practices and recognizing and managing symptoms when they occur [8, 9]. Self-care is composed of three dimensions: health promotion and treatment adherence (maintenance), body listening and symptom recognition (monitoring), and taking action to manage signs and symptoms (management) [8]. Figure 1 shows the conceptual framework of the Mid-Range Theory of Chronic Disease Self-Care integrating symptoms with self-care [9].

The Self-Care of Chronic Illness Inventory (SC-CII) [2] was designed to measure each of these self-care processes using three separate scales measuring Self-Care Maintenance, Self-Care Monitoring and Self-Care Management. After demonstrating content validity, psychometric tests were conducted, concluding that the SC-CII was adequate in reliability and validity. The conclusions of the study suggested the need for further testing in diverse populations of patients with chronic diseases.

However, the SC-CII is a generic instrument developed to measure self-care behaviors, and it is possible that cultural beliefs may influence self-care behaviors and the interpretation of the measure. To explore this further, the equivalence of the SC-CII measurement was investigated in individuals from three different cultural groups: Italians, Swedes and Americans [3]. The findings of this cross-cultural assessment of the SC-CII determined that patients in all three countries used an identical cognitive framework or mental model when responding and used the Likert 1–5 response scale in an almost identical and unbiased manner. Despite sociocultural differences, patients in these countries shared the same fundamental view of self-care, so the results of the SC-CII were comparable across these countries [3], which boosts the generalizability of research findings on self-care of chronic illness using the SC-CII. This cross-cultural study indicated the need for further research in other cultural and national contexts to determine whether the construct of self-care that is common in these three countries is also valid for other populations. Studies of patients in different sites could increase the generalizability of these findings. In psychometric literature, measurement invariance indicates that the same construct is being measured across groups [3].

In this context, and considering the current development of the SC-CII, the purpose of this study was to cross-culturally adapt the SC-CII, test its psychometric properties and validate its use among Spanish people with chronic diseases. This will allow us to determine whether there is a shared construct of self-care by equivalence of measurement noted in the cultural contexts of previous studies that point towards a shared mental model, although minor differences in specific behaviors can be identified.

## Methods

### Study design

To conduct this investigation, we performed a multicenter cross-sectional study to describe self-care in patients with multiple chronic illnesses.

### Sample/participants

A total of 350 Spanish outpatients and inpatients were recruited in ten sites in western and southern Spain. All fulfilled the inclusion and exclusion criteria. Inclusion criteria were: aged 18 or over, and having at least one chronic illness. No specific disease or disease stage was targeted for enrolment. Exclusion criteria were dementia or inability to read and write in Spanish.

### Data collection and instruments

Data for the present study were collected from face-to-face interviews during 2022. Identification of the cases and data collection at each site was done at outpatient or inpatient level. Inclusion and exclusion criteria were then revised, and the programmed activity for each patient was reviewed with a view to their participation in the study when they attended for their next appointment if they were outpatients. Inpatients were visited in the corresponding unit during their recovery. Interviews were conducted by research assistants who were all nurses trained in the study protocol. When the research assistants met with potential participants, they explained the study aims and asked them to sign the informed consent form. Once the informed consent was signed, patients were asked to complete all study instruments administering the SC-CII with a brief demographic and clinical questionnaire. Survey completion took approximately 15 min. SC-CII was completed twice by a sample of 60 patients to calculate test–retest reliability. For the purposes of the present study, the followings instruments were considered.

A clinical interview was used to assess self-reported sociodemographic data, and clinical variables of the patients. To collect socio-demographic characteristics, a structured questionnaire was developed by our research team. Researchers collected data relating to age, gender, marital status, number of children, education level, employment and income. Patients’ clinical histories were used for collecting clinical characteristics, including the number and type of chronic illnesses.

The SC-CII is a generic measure of self-care [2] that was designed for use in individuals with any number and type of chronic conditions. The 19-items of the SC-CII are divided among three separate scales measuring the three behavioral processes. The 7-item Self- Care Maintenance scale had two factors: “health promoting behavior” (items 1, 3 and 7) and “illness related behavior” (items 2, 4, 5 and 6). The 5-item Self-Care Monitoring scale had a single factor (items 8–12). The 7-item Self-Care Management scale had two factors: “autonomous behavior” (items 13, 14, 15 and 19) and “consulting behavior” (items 16, 17 and 18).

Self-care maintenance reflects primarily health promoting and maintenance behaviors such as exercise and taking medication as prescribed. Self-care monitoring involves checking oneself for changes in signs and symptoms. Self-care management reflects the response to changes in signs or symptoms when and if they occur (e.g. adjusting diet or medication based on detection and interpretation of symptoms) [3].

All items are rated on a 5‐point ordinal response scale. The Self‐Care Maintenance and Self‐Care Monitoring scales ask: “How often do you do the following things?”. Responses range from never to always. The Self‐Care Management scale asks: “How likely are you to use one of these actions?” Responses range from not likely to very likely. Two items in the Self‐Care Management scale include a 0 option (i.e., “I did not recognize the symptom”; “I did not do anything to manage symptoms”). Each of the three scales was scored separately and standardized from 0 to 100 with higher scores indicating better self-care. A cut-off point of 70 was used to reflect adequate self-care [10].

Initial psychometric testing revealed content validity and adequate reliability of the three scales [2]. Also, the SC-CII demonstrates an excellent level of invariance and good psychometric properties in different populations [3].

### Translation procedure

After obtaining permission from the original author and completing an Instrument Translation Agreement, SC-CII was translated into Spanish following the recommended guidelines for cross-cultural adaptation [11], the steps recommended by the authors of the instrument [12] and the translation procedure used in the psychometric evaluation of other previously validated self-care instruments such as in the cross-cultural adaptation conducted by Chen et al. [13].

Forward translation. First, two independent bilingual translators translated the SC-CII into Spanish. Both of them were fluent in English and Spanish with extensive experience in the process of translation and back-translation of questionnaires.

Synthesis of the two translated versions. After the forward translation, a comparison between the two translated Spanish versions of SC-CII and the original scale was made by a third translator. The study researchers discussed and resolved all ambiguities and inconsistencies. A consensus version was obtained.

Back translation. Two independent translators with extensive experience in the process of translation and back-translation of questionnaires translated the Spanish version of SC-CII back into English.

Synthesis of the two back-translated versions. Our research team clarified the wording, grammatical structure, meaning equivalence and relevance of the two back-translations. Some ambiguities and inconsistencies were referred to the translators for clarification.

Assessment of cultural equivalence. The Spanish translated version of the scale and its respective back-translations were compared with the original version. This process was carried out by a committee of experts (5 nurses and 1 doctor) with extensive experience in the field of chronic diseases (minimum 10 years) who followed the criteria established by the European Research Group on Health Outcomes [14] to assess the concordance of the translations.

Pilot testing and comprehensibility survey. The prefinal SC-CII Spanish version was pilot tested using a sample of 80 patients. Participants took between 9 and 10 min to fill out the scale. In particular, each participant was invited to use ‘clear’ or ‘unclear’ to describe the instructions and items of the scale, and was asked to provide suggestions on how to make each item clearer, a procedure similar to that followed by previous studies in the process of adapting and validating self-care assessment instruments [15]. After all the procedures, the final SC-CII Spanish version was generated for psychometric evaluation.

### Data analysis

Data were analyzed by SPSS 27 and the R-package Lavaan. Both sociodemographic variables and the scale itself were first studied from a descriptive point of view. Distribution of each subscale was analyzed after standardization (0 means minimum possible score and 100 maximum).

A confirmatory factorial analysis (CFA) was carried out in order to evaluate whether the structures found in the original scale fit the data. The SC-CII was developed according to the Middle-Range Theory of Chronic Illness Self-Care, and three dimensions of the scale were determined. Therefore, CFA was employed to test construct-related validity. The sample size of 100–400 was considered adequate, and 200 was considered most appropriate for CFA [15]. To approximate previous validation studies based on the middle-range theory of self-care of chronic illness [2, 15–19], CFAs were carried out on three scales of the SC- CII. To estimate the model we chose in every case the best option between the Maximum Likelihood method (ML) and robust Maximum Likelihood (MLR) [20], due to the strong skewness found in our variables. To examine model fit we considered several measures: the Comparative Fix Index (CFI), the Tucker and Lewis Index (TLI), the Root Mean Standard Error Approximation (RMSEA), with corresponding 90% Confidence Interval (CI) and p-value, and the Standarized Root Mean Square Residual (SRMS). The χ2 likelihood ratio test was also applied, given its sensitivity to sample size and model hypothesis violations. We aimed to obtain CFI and TLI over 0.90, SRMR and RMSEA under 0.08 and p-value less than 0.05 for the χ2 test. Factor loadings should be significant. Correlations between factors were analyzed by means of Pearson’s r test. To assess internal consistency, McDonald’s ω was calculated rather than Cronbach’s α when unidimensionality was not found. Values over 0.70 were considered acceptable in both cases. The intraclass correlation coefficient (ICC) was employed to assess test–retest reliability. One-week to two-week intervals were recommended to measure test–retest reliability [21], and so the same scale was completed again two weeks later, by only 60 subjects of the total sample, in order to evaluate test–retest validity through the ICC. 60 participants were enough to detect an effect size δ = 0.50 with β = 0.10 and α = 0.05 by t-paired test. Analyses were made for each scale separately before proceeding to the global analysis of the model.

## Results

### Translation and cultural adaptation

After the process of translation and back-translation of the SC-CII, a first version translated into Spanish was available, the product of 2 independent translations and 2 independent back-translations of this first consensual translation.

In order to check whether the Spanish-translated version would be assessed in the same way as the original version, a committee of experts compared the original English version with the consensus version translated into Spanish and the two back-translations into English. To do so, they determined the category to which each item belonged according to the European Research Group on Health Outcomes [14] (84% to group A [conceptually equivalent], 16% to group B [although some words change, the final meaning does not change], 0% to group C [loss of the meaning of the item]). Given that none of the items had lost their original meaning after the translation and back-translation process, it was accepted that the version translated into Spanish had the optimal semantic equivalence to conclude this phase.

Finally, we conducted a comprehensibility survey in 80 patients to check whether the scale was adequately understood and could be filled in correctly. The selection of these patients was done on a random basis.

Pre-test data showed easy comprehension of the items. The time taken to complete the scale ranged from 9 to 10 min. The following terms were amended in the SC-CII: in items 17 and 18, “healthcare provider” was not easily understood in Spanish culture and it was replaced with “doctors or nurses”. Furthermore, the phrase “take prescribed medicines” was changed to “take medicines prescribed by the doctor” in item 8. The origin SC-CII scale, and the translated and adapted version, are shown in Table 1.

**Table 1:** The original and final versions of Self-Care Chronic Illness Inventory scale items

Original SC-CII scale items	Translated and adapted version
1.Make sure to get enough sleep	1.Ensure enough sleep 1.Asegurarse de dormir lo suficiente
2. Try to avoid getting sick (e.g., flu shot, wash your hands)	2. Try to avoid the risk of disease (e.g. get vaccinated against the flu, handwashing) 2. Intentar evitar el riesgo de enfermedad (p. ej., vacunarse contra la gripe, lavarse las manos)
3. Do physical activity (e.g., take a brisk walk, use the stairs)	3. Attend physical activity (e.g., take a brisk walk, use the stairs) 3. Realizar actividad física (p.ej., dar un paseo a buen paso, subir por las escaleras)
4. Eat special foods or avoid certain foods	4. Follow a special diet or avoid certain foods 4. Seguir una dieta especial o evitar ciertos alimentos
5. Keep appointments for routine or regular health care	5. Keep appointments for routine or regular health care 5. Acudir a las citas médicas de forma rutinaria o regular
6. Take prescribed medicines without missing a dose	6. Take prescribed medicines by the doctor and never miss a dose 6. Tomar la medicación que le ha recetado su médico sin saltarse ninguna toma o dosis
7. Do something to relieve stress (e.g., mindfulness, yoga, music)	7. Do something to ease stress (e.g., mindfulness, yoga, listening to music) 7. Hacer algo para aliviar el estrés (p.ej., meditación, yoga, escuchar música)
8. Monitor your health condition	8. Monitor your health condition 8. Evaluar su estado físico
9. Monitor for medication side-effects	9. Evaluate the side-effects of your medication 9. Evaluar los efectos secundarios de su medicación
10. Pay attention to changes in how you feel	10. Pay attention to changes in how you feel 10. Prestar atención a si hay cambios en cómo se siente
11. Monitor whether you tire more than usual doing normal activities	11. Assess if you get more tired than usual when doing everyday activities 11. Evaluar si se cansa más de lo habitual realizando actividades cotidianas
12. Monitor for symptoms	12.Monitor for symptoms 12. Evaluar sus síntomas
13. How quickly did you recognize it as a symptom of your health condition?	13. How quickly did you recognize it as a symptom of your health condition? 13. ¿Con qué rapidez notó usted que se trataba de un síntoma de su enfermedad?
14. Change what you eat or drink to make the symptom decrease or go away	14. Change what you eat and drink so that the symptom lessens or disappears 14. Cambiar lo que come y bebe para que el síntoma disminuya o desaparezca
15. Change your activity level (e.g., slow down, rest)	15. Change your activity level (e.g., slow down, rest) 15. Variar su nivel de actividad (p.ej., reducirlo, descansar)
16. Take a medicine to make the symptom decrease or go away	16. Take a medicine to make symptoms lessen or disappear 16. Tomar medicación para que el síntoma disminuya o desaparezca
17. Tell your healthcare provider about the symptom at the next office visit	17. Tell your doctor or nurse about your symptoms at the next visit 17. Hablar sobre sus síntomas con su médico o enfermera en la próxima visita
18. Call your healthcare provider for guidance	18. Call your doctor or nurse for guidance 18. Llamar a su médico/enfermera para que le aconseje
19. Did the treatment you used make you feel better?	19. Did the treatment you used make you feel better? 19. ¿El tratamiento que siguió la última vez le hizo sentir mejor?

### Sociodemographic and clinical characteristics of participants

The sociodemographic and clinical characteristics are summarized in Table 2. In total, we enrolled a sample of 350 patients. Most (63.4%) participants were older adults aged 65 years or older with a mean age of 65.45 ± 14.97 that ranged from 22 to 88 years, with at least some college education (32.9%). About 20% were employed full or part‐time and 41.4% reported an annual income sufficient to make ends meet. The average number of chronic conditions reported was 2.81%; the most common conditions were hypertension (52.3%), musculoskeletal disorders (e.g., arthrosis, arthritis) (46.3%) and diabetes (38.9%). Overall, more women than men were enrolled.

**Table 2:** Sociodemographic data and characteristics of the sample

Sociodemographic characteristics				
**Variable**	**Categories**	**N (%)**	**Mean ± SD**	**Median,range**
Age (years)			65.45 ± 14.97	(69,68)
Gender	Male Female	171 (48.9) 179 (51.1)		
Marital status	Single Married or partnered Divorced or separated Widowed	46 (13.1) 224 (64) 27 (7.7) 53 (15.1)		
Number of children			2.14 ± 1.19	(2,4)
Education level	No studies Elementary school Middle school High school Higher education	59 (16.9) 115 (32.9) 30 (8.6) 60 (17.1) 86 (24.6)		
Employment status	Currently in employment Temporary sick leave Permanent sick leave Unemployed Retired	69 (19.7) 6 (1.7) 30 (8.6) 43 (12.3) 202 (57.7)		
Income	Comfortable; have more than enough to make ends meet Have enough to make ends meet Do not have enough to make ends meet	124 (35.4) 145 (41.4) 81 (23.1)		
**Clinical characteristics**				
**Variable**		**N (%)**	**Mean ± SD**	**Median,range**
Total number of chronic illnesses			2.81 ± 1.64	(2,9)
Heart failure		24 (6.9)		
Diabetes		136 (38.9)		
Hypertension		183 (52.3)		
Neurological disorder (stroke, paralysis, dementia…)		32 (9.1)		
Pulmonary disease (asthma, emphysema, lung disease…)		57 (16.3)		
Kidney disease		12 (34)		
Musculoskeletal disorders (e.g., arthrosis, arthritis…..)		162 (46.3)		
Gastrointestinal disease		54 (154)		
Liver disease		9 (26)		
Oncological problems (skin cancer, breast cancer, prostate cancer…)		40 (11.4)		
Other heart problems (arrhythmia, congenital heart disease, atrial fibrillation…)		24 (69)		
Other diseases (thyroid problems, vascular problems…)		30 (86)		

*Abbreviations*: *SD* Standard deviation

### SC-CII items description

Table 3 reports the descriptive statistics of the SC-CII items: mean, standard deviation, skewness and kurtosis. The items with the highest score were item 6 “Take prescribed medicines by the doctor and never miss a dose?” and, with the same average, item 5 “Keep appointments for routine or regular health care?” and item 12 “Monitor for symptoms?”. The items with the lowest score were item 4 “Follow a special diet or avoid certain foods?” and item 1 “Ensure enough sleep?”. Regarding item 13, “How quickly did you recognize it as a symptom of a health condition?”, only 10.6% (*n* = 37) of participants did not recognize the symptoms. Of those participants who did recognize it (84.6%; *n* = 241), 5% (*n* = 12) did not recognize it quickly, 26.5% (*n* = 64) recognized it somewhat quickly and 53.1% (*n* = 128) recognized it very quickly.

**Table 3:** Descriptive statistics of the SC-CII items

**Subscale**	**Item**	**Mean** ± SD	**Skwedness**	**Kurtosis**
**Self-Care Maintenance** **(*N* = 350)**	Item 1	3.510 ± 1.214	-0.469	-0.469
	Item 2	4.706 ± 0.751	-3.424	-3.424
	Item 3	3.936 ± 1.141	-0.843	-0.843
	Item 4	3.083 ± 1.633	0.020	0.020
	Item 5	4.750 ± 0.696	-3.209	-3.209
	Item 6	4.868 ± 0.618	-5.213	-5.213
	Item 7	3.828 ± 1.447	-0.687	-0.687
**Self-Care Monitoring** **(*N* = 350)**	Item 8	4.71 ± 0.695	-2.974	-2.974
	Item 9	4.451 ± 1.171	-2.232	-2.232
	Item 10	4.740 ± 0.592	-2.508	-2.508
	Item 11	4.627 ± 0.650	-1.735	-1.735
	Item 12	4.750 ± 0.629	-2.732	-2.732
**Self-Care Management** (*N* = 232)*	Item 13	4.216 ± 1.163	-1.340	0.941
	Item 14	3.621 ± 1.163	-0.644	-1.061
	Item 15	3.975 ± 1.276	-1.107	0.194
	Item 16	3.924 ± 1.463	-1.016	-0.453
	Item 17	4.652 ± 0.790	-2.289	4.204
	Item 18	3.934 ± 1.508	-1.044	-0.496
	Item 19	3.842 ± 1.640	-1.085	-0.303

^*^Items 14–18 were only completed by subjects who answered item 13. *Abbreviations*: *SD* Standard deviation

**Fig. 2:**
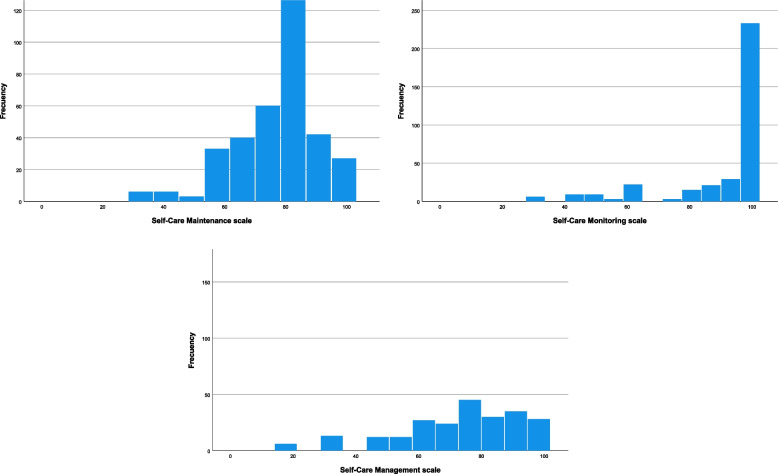
SC-CII scales histograms

Figure 2 displays histograms obtained from our sample which show how the scales performed. Each scale was previously standarized so that the minimum possible score was 0 and the maximum 100. The Self-Care Maintenance scale average was 76.93 ± 13.78; the Self-Care Monitoring scale average was 91.30 ± 16.90, and the Self-Care Management scale average was 75.74 ± 17.86.

### Structural validity and reliability analysis

This analysis was made separately for each scale and then globally.

#### Self-care maintenance scale

The original study proposed a bifactorial structure for this first scale: one factor “health promoting behavior” consisting of item 1 (sleep), item 3 (physical activity) and item 7 (stress management), and another factor “illness related behavior” consisting of item 2 (avoid sickness), item 4 (special diet), item 5 (routine health care) and item 6 (take medicines). In our case, the CFA for this structure provided CFI = 0.923, TLI = 0.876, SRMR = 0.059, RMSEA = 0.069, 90% CI = 0.042- 0.097 (*p* = 0.017), χ^2^ = 34.667 (*p* < 0.001). Nevertheless, inspection of the modification indices revealed that item 4 loaded on “health promotion behavior”. Moreover, we found an unacceptable factorial loading of 0.081 for item1 (sleep) due to the lack of correlation between this item and the rest. This issue was not reported in the original version but led us to consider two possible models: a model regarding item 1 itself as a factor, or a model without item 1. When we re-specified the model with item 4 loaded in the “health promoting behavior” factor and the removal of item 1, the fit of the model improved: CFI = 0.979, TLI = 0.960, SRMR = 0.034, RMSEA = 0.046, 90% CI = 0.000–0.085 (*p* = 0.495), χ^2^ = 13.815, *p* = 0.087. All the factor loadings were significant.

**Fig. 3:**
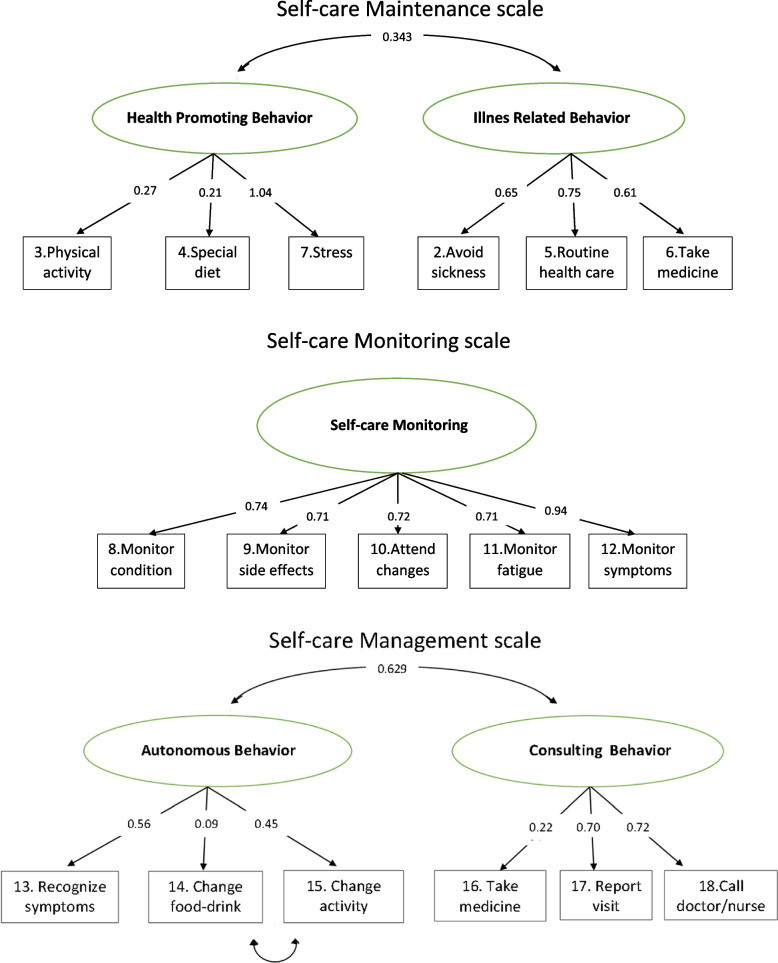
Graphic representation of the CFA of the SC-CII scales

Therefore, although we consider acceptable the reduced structure proposed for the original English version, the model in Fig. 2 might be more appropriate, at least for the Spanish version. Factor loadings are shown in Fig. 3. All of them are significant. Correlation between the factors was significant (*r* = 0.343, *p* < 0.001). Internal consistence analysis provides as a result ω = 0.757.

#### Self-care monitoring scale

The original study proposed a unidimensional model for this scale (from items 8 to 12). In our case robust CFA provided similar results: CFI = 0.981, TLI = 0.962, SRMR = 0.029, RMSEA = 0.036, 90% CI = 0.000–0.088 (*p* = 0.602), χ^2^ = 7.252, p = 0.203. Factor loadings are shown in Fig. 3. All of them are significant. Internal consistency measure was α = 0.856.

#### Self-care management scale

This scale is also evaluated if the subject presents any symptoms. The sample size was N = 232. The original study considered a bifactorial structure. Self‐Care Management is defined by the two dimensions of “autonomous behavior” (items 13, 14, 15 and 19) and “consulting behavior” (items 16, 17 and 18). CFA testing with these two factors yielded a poor fit: CFI = 0.661, TLI = 0.453, SRMR = 0.087, RMSEA = 0.100, 90% CI = 0.068–0.134 (*p* = 0.007), χ2 = 43.367 (*p* < 0.001). An inspection of the modification indices revealed that the main cause of misfit was attributing item 19 (Did the treatment you used make you feel better?) to both factors 1 and 2. The re-specification of the model without item 19 improved the model fit. We considered item 19 to be confusing for the reasons explained below. After analyzing all the possibilities, the best fit corresponded to a bifactorial structure (without item 19). CFA showed a first factor (autonomous behavior), consisting of items 13, 14 and 15, (considering residual covariances between items 14 and 15) and a second factor (consulting behavior) consisting of items 16, 17 and 18. Results of robust CFA were acceptable: CFI = 0.878, TLI = 0.738, SRMR = 0.052, RMSEA = 0.078, 90% CI = 0.031–0.127 (*p* = 0.141), χ2 = 16.95 (*p* = 0.018). Factor loading of item 14 was low (*p* = 0.417). Both factors were correlated (*r* = 0.629, *p* < 0.001). The model is illustrated in Fig. 3. As a measure of its internal consistency, we obtained ω = 0.726.

#### Global model

We obtained our model by simply adding the five factors found in the previous analyses. Results were: CFI = 0.762, TLI = 0.700, SRMR = 0.090, RMSEA = 0.056, 90% CI = 0.042–0.069 (p = 0.229), χ2 = 186.23 (*p* < 0.001). Measure of its internal consistency was ω = 0.931. All factor loadings were significant except for item 14 (*p* = 0.051). Correlation between factors are shown in Table 4. We highlight the strong correlation (*r* = 0.882) between the “illness related behavior” factor and the Self-Care Monitoring scale.

**Table 4:** Simultaneous CFI of the SC-CII factors

		Health promoting behavior	Illness related behavior	Self-care Monitoring	Autonomous behavior	Consulting behavior
Self-Care Maintenance	Health promoting behavior					
	Illness related behavior	0.413*				
Self-Care Monitoring		0.362*	0.882*			
Self-Care Management	Autonomous behavior	0.532*	0.649*	0.373*		
	Consulting behavior	0.090	0.253*	0.328*	0.087	

^*^(*p* < 0.05)

Finally, for test–retest reliability, after 2 weeks, 60 patients completed the follow-up assessment. Test–retest reliability showed the range of ICC was between 0.89 and 0.92, indicating that the three scales of SC-CII have satisfactory stability.

## Discussion

The fact that we live in a society and an age in which the exchange of information is essential for progress in all fields of science means that assessment instruments generated in one country are quickly used in another. This has created the need for such instruments to be comparable across countries, which requires having accessible assessment tools that are culturally adapted to each country. The purpose of this study was to test the psychometric properties of the SC-CII in the Spanish population. The recommendations for each of the stages of the translation process and cultural adaptation of the scale into Spanish have been respected [11, 12]. In addition, as recommended, the translated version was submitted to a population equivalent to that used to construct the original version [2], respecting the variability of diagnoses and the age range of the population, as well as similar populations studied in the cross-cultural assessment of the SC-CII [3].

SC-CII is a measure based on self-care theory designed for use in individuals with chronic diseases, regardless of diagnosis. The authors of the questionnaire [2] hypothesized that it would be a valuable generic instrument for measuring self-care in populations with more than one chronic disease and useful for comparing self-care behaviors in different populations and interventions, an aspect that is corroborated in our study although with small differences in the dimensionality of the items of the scales of this instrument, which will be explained below.

Regarding the standardized mean scores obtained on the three scales, previous research using self-care instruments based on the same theory has suggested that a cut-off point of ≥ 70 would be indicative of adequate self-care [10]. However, in previous studies, this score indicated adequate behaviors on the Self-Care Maintenance and Monitoring scales, but not on the Self-Care Management scale [22, 23]. In contrast to these results, in our sample, adequate self-care behaviors were obtained in all three scales of the SC-CII (all above the recommended threshold of 70), including Self-Care Management, although its mean score is the lowest of the three. This can be explained, in contrast to other populations, by a greater demand for health care, the rise of primary care in Spain, the fact that the Spanish population is more dependent on health care services and a health care model based on continuity of care rather than convenience, and without pronounced financial barriers compared to other health systems (e.g. in the United States). In our study, patients performed better in Self-Care Monitoring than Self-Care Maintenance or Management, in line with other research [24], although we find it interesting to note that the average obtained was significantly higher [22–24]. Previous studies have demonstrated that patients with a single chronic condition performed self-care monitoring practices; however, these studies did not examine the other self-care behaviors of maintenance and management [25]. In our context, this increased self-care monitoring behavior can be explained by the primary care approach or model in our country that encourages and promotes health education and patient self-management and follow-up, including the provision of monitoring devices on an increasing basis, which improves motivation and collaboration between patient and health professional and the availability of clinical data.

Initial psychometric testing in the Spanish population shows that the instrument can be used on people with one or more chronic illnesses. We found satisfactory reliability and construct validity mainly in the first two scales, with adequate results in the third (Self-Care Management), by using residual correlations. It could be considered controversial but acceptable from a theoretical point of view [26]. It probably points to the need to reconsider the dimensionality of the same items in future studies. However, we can be sure that there are differences in self-care that are identified when results are compared with other populations and cultures, as previous results point out [3]. We interpret the fact of differences in measurement in heterogeneous populations as an indicator of the sensitivity to change of the SC-CII. Even if the factorial structure of the three scales were confirmed by the CFA and is coherent with those of previous self-care instruments [2, 3], a modification in the dimensional structure of the items would lead to an excellent fit of the model for our population, which we would have to test in further studies.

### Self-care maintenance scale

Following the original study, in terms of the dimensionality of the Self-Care Maintenance scale, there are items related to both “health promoting behavior” and “illness related behavior”, which was explained by Riegel et al. [2], who considered that in order to improve well-being, preserve health or maintain physical stability and emotional stability (self-care maintenance), behaviors aimed at both promoting health (e.g. getting enough sleep) and coping with illness (e.g. taking medication) contribute to this concept.

We found that the model performed better when item 1 “Ensure enough sleep” was excluded. Despite the fact that sleep/rest is a basic human need or self-care, it is not given due attention in our environment. The differences found in the item 1 could be explained by the influence of cultural factors on the attitude to sleep of the Spanish population. Various conditioning factors such as climate, traditions and the availability of light contribute to differences in sleep patterns in different populations, in addition to the influence that the disease may have on them [27].

In the initial development and testing of the SC-CII [2], item 4 "Follow a special diet or avoid certain foods" was found to be an “illness related behavior” and not a “health promoting behavior”. This same dimensionality was proposed by De Maria et al. [3] when they conducted cross-cultural assessment of the SC-CII in three different samples of patients (Italians, Swedes, and Americans). In contrast, our results find that item 4 is linked to the “health-promoting behavior” dimension, i.e. people in our sample seem to see healthy eating as a choice rather than a necessity, suggesting that people have been successfully engaged through appropriate health education in early healthy eating before food preferences are set.

### Self-care monitoring scale

The Self-Care Monitoring scale was described as unidimensional, comprising five items, from 8 to 12. Our results provided a perfect fit of this model in line with the results provided above [2, 3]. These results demonstrate that Spanish people have the same conceptual definition of self-care monitoring and use the same behaviors, such as monitoring for symptoms, health condition, fatigue, medication side-effects and changes in health status as other analyzed population groups.

### Self-care management scale

In testing the self-care management scale, we found a problem with the original model proposed. This scale is described as having the two dimensions of “autonomous behavior” and “consulting behavior”, measured by four and three items, respectively. Thus, we specified a two-factor model CFA. We hypothesized that items 13, 14, 15 and 19 were loading the “autonomous behavior” factor, and items 16, 17 and 18 the “consulting behaviors” factor, following initial testing of SC-CII [2], despite the fact that subsequently when De Maria et al. [3] conducted the cross-cultural psychometric assessment of the inventory, they concluded that this posited model showed an inadequate fit. They found that the main cause of misfit was attributing item 16 (“take medicine to get symptoms lessens or disappeared) to factor 2 (consulting behavior). The re-specification of their model with item 16 attributed to factor 1 (autonomous behavior) in all three countries studied was supported by excellent fit indices. As in the original study, in our population, in the factor structure the fit index improves when this item loads on factor 2 (consulting behavior). The meaning of this item is also congruent with autonomous behavior, another factor that refers to the capability of the patient to recognize symptoms, to change eating and drinking habits and activity level. Although, the fact of including the item 16 "take a medicine to make symptoms lessen or disappear” within the dimension "autonomous" or derived from a "consulting behavior" is complex due to the meaning of the concept of self-medication. Self-medication is the selection and use of medicine (including herbal and traditional products) by individuals to treat self-recognized illnesses or symptoms. Self-medication is one element of self-care [28]. Nevertheless, responsible self-medication is the use of a registered or monographed medicine legally available without a physician’s prescription, either on an individual’s own initiative or following the advice of a healthcare professional. The World Medical Association in its statement on self-medication proposes a clear differentiation between self-medication and prescription-based medication [29]. The use of prescription medicines without a prior medical prescription is not part of responsible self-medication [29]. That is, the consumption of medications for chronic diseases, which in most cases are prescription drugs, will require a medical consultation. Although this has been changing in recent years, it is true that traditionally self-care and self-medication were regarded as unnecessary and potentially even unhealthy practices. This paternalistic approach to medicine, supported by health systems designed to treat sickness (rather than to prevent disease) remains a familiar aspect of health care in many countries to this day [30]. Nevertheless, self-medication represents a complex problem, with aspects related to the population, such as medical education, culture or other factors.

Moreover, item 19 was not included in the analysis of this scale. We believe that item 19 can be confusing. This item refers to whether the treatment used made you feel better, i.e. it is focused differently to the other items of the Self-Care Management scale. The rest of the items of the scale (items 14–18) refer to the actions that patients took to improve their symptoms, while item 19 refers cross-sectionally to whether these actions had an effect on their symptoms.

As the original study, we found a strong correlation between “illness related behavior” and “self-care monitoring”. Whether this issue is due to a moderate fit for the global model or a superposition of both factors is not clear. However, we can think of interoceptive awareness as a dimension underlying both illness-related behavior and self-care monitoring. Monitoring or interoceptive awareness, i.e., the relationship between the value judgment of interoceptive performance (interoceptive awareness) and the patient's actual performance (monitoring) may be closely related.

### Limitations

Our study is not without limitations. First, our study is limited by the choice of the convenience sampling, which may not be sufficiently representative. Second, the cross-sectional nature of the study is a limitation, so future studies will need to possibly verify the construct validity longitudinally. On the other hand, self-care was self-reported, which might not reflect actual self-care behaviors. Finally, it is impossible to check the validity of the criteria, as there is no other self-care assessment scale for chronic diseases that has completed the validation process for the Spanish population, and it is not possible to compare the results with another instrument that measures the same construct. A strength of the study is that it is the first to describe self-care maintenance, self-care monitoring and self-care management in patients with chronic illness in Spain. Future studies sampling patients from different sites in our country or in other Spanish speaking ones may increase the generalizability of our findings.

## Conclusions

The reliability analysis provided acceptable results. The model structure analysis provided a good fit for the Self-Care Maintenance and Self-Care Monitoring scales and an acceptable fit for Self-Care Management and the global model. We consider the Spanish version of the SC-CII to be psychometrically equivalent to the original version in terms of its validity and reliability, which supports its usefulness in assessing self-care in the Spanish adult population with chronic illness both in clinical practice and in research. The adapted version of the SC-CII has been shown to possess the instrumental properties of its original version with only some differences in the dimensionality of some items when adapted to our country and culture (model with item 4 “special diet” loaded in the “health promoting behavior” factor and the removal of items 1 “sleep” and 19 “evaluate action”). The evidence of validity and reliability supports its use. The scale provides a simple and rapid solution to assess the self-care process carried out by patients, which is important given the increasing rate of chronic diseases, self-care being an essential element of their care.

### Implications for nursing and health policy

The establishment and accuracy of a tool, which although it may be sensitive to ethnic and cultural differences of patients, demonstrates that the concept of self-care is common in different countries and is also valid for culturally diverse populations. This study contributes to a common self-care assessment framework can be used by nurses when defining interventions focusing on self-care according to the patient mastering and management, thus better patient outcomes and quality of care. Many adults have more than one chronic condition, so a generic measure of self-care would allow measurement of self-care in these complex populations with chronic conditions. The Self-Care Chronic Illness Inventory can help to identify people or populations who have decreased self-care and, hence, poor health outcomes, which will help nurses to tailor health education for their patients. To counteract the burden of chronic illnesses, healthcare systems, providers and policy makers must promote patient self-care.

## Data Availability

The data sets used and analysed during the current study are available from the corresponding author on reasonable request.

## Declarations

### Permissions

Permission was requested and obtained from the author of the SC-CII to create and validate this Spanish-language version.
